# Benign teratoma of the liver: a rare cause of cholangitis

**DOI:** 10.2349/biij.2.3.e20

**Published:** 2006-07-01

**Authors:** K Rahmat, A Vijayananthan, BJJ Abdullah, SM Amin

**Affiliations:** 1 Department of Biomedical Imaging, Faculty of Medicine, University of Malaya, Kuala Lumpur, Malaysia; 2 Department of Surgery, Faculty of Medicine, University of Malaya, Kuala Lumpur, Malaysia

## Abstract

Teratomas are neoplasms characterised by an abnormal growth of tissues derived from the three germinal layers. The term ‘teratoma’ is derived from the Greek root ‘teratos’, meaning monster. Germ cells develop in the embryo and subsequently become the cells that make up the reproductive system. During fetal development, these cells follow a midline path and descend into the pelvis as ovarian cells or the scrotal sac as testicular cells. The presence of germ cells in extragonadal sites is because of the failure of these cells to migrate along the urogenital ridge. Therefore, teratomas occur in order of decreasing frequency in the ovaries, testes, anterior mediastinum, retroperitoneum, sacrococcygeal region and cranium.

Liver teratomas are very rare; of the 25 hepatic teratomas described in the literature, only five have occurred in adults. The majority of the cases were in female children below the age of three, mostly arising in the right lobe of liver.

We report a case of an adult male with benign mature teratoma arising in the left lobe of liver, compressing the common bile duct and causing obstructive jaundice.

## CASE REPORT

A 46-year-old man was admitted for progressive jaundice and pyrexia occurring arbitrarily for a month. He also complained of episodes of chills and rigors, and showed significant weight loss. The patient was passing pale coloured stools and had pruritus. He also had a history of vomiting associated with upper abdominal pain. There was no history of bleeding tendencies, and the patient claimed to be non-alcoholic. His previous medical history was normal.

On clinical examination, he revealed jaundice with scratch marks seen on the skin of the arms and abdomen. His urine was dark coloured, and there was a palpable tender firm epigastric mass extending to the right hypochondrium.

The blood examination was consistent with obstructive jaundice: total bilirubin 99 mmol/L (3-17 mmol/L) conjugated bilirubin 93 mmol/L (0-3 mmol/L), alanine transaminase 127 IU/L (30-65 IU/L), aspartate transaminase 50 IU/L (15-37 IU/L), alkaline phosphatase 196 IU/L (50-136 IU/L) and GGT 124 IU/L (15-85 IU/L). A full blood count showed a haemoglobin level of 127 g/L, white blood cell count of 11.7 x 10^9^ and platelet count of 320 x 10^9^. Hepatitis serology was negative, and the diagnosis was obstructive jaundice secondary to possible pancreatic malignancy.

An ultrasound examination revealed a heterogenous solid mass measuring 5.0cm x 6.5cm x 8.0cm with echogenic areas in the epigastrium, medial to the gallbladder. The left lobe of liver was atrophic, and the intra and extra hepatic biliary ducts were of normal calibre.

**Figure 1:**
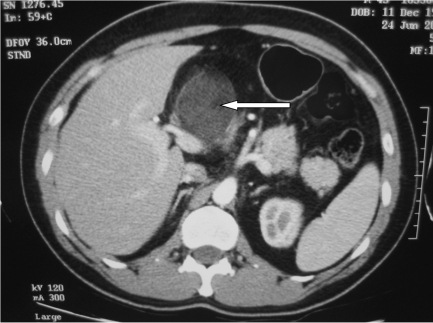
Contrast enhanced CT scan of the abdomen showed a heterogeneous soft tissue mass (white arrow) in the anterior quadrant, lying anterior to the portal vein.

**Figure 2:**
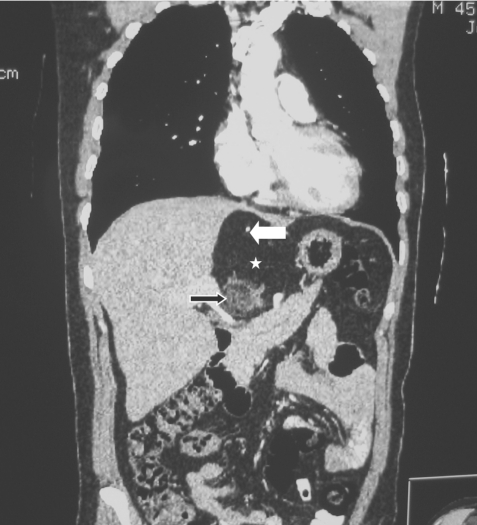
Coronal CT scan of the abdomen. The mass showed combination of fat (asterisk), calcification (white arrow) and soft tissue (black arrow).

A contrast enhanced computed tomography (CT) examination (Figures 1 and 2) revealed a heterogeneous mass in the anterior upper quadrant, located medial to the left hepatic lobe, anterior to the main portal vein and the head of pancreas. This mass consisted of dense calcification, fat and soft tissue, and compressed the common bile duct (CBD). The CBD was dilated and the lobulated lesion was clearly demarcated from the atrophic left lobe of liver and the porta hepatis. This mass was diagnosed to be a teratoma of either peritoneal or hepatic origin.

**Figure 3:**
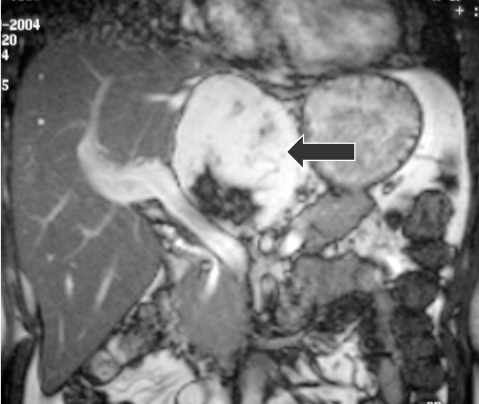
Magnetic Resonance Imaging (MRI) sequence (coronal view). The mixed signal intensity mass (black arrow) appears separate from the portal vein and the arterial branches.

**Figure 4:**
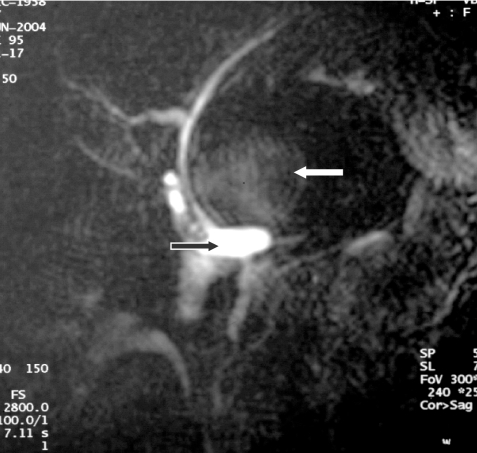
Magnetic Resonance Cholangiopancreatography (MRCP). There is dilatation of the common bile duct (black arrow) caused by compression of the mass (white arrow) at its distal part. No communication was seen between this mass and the biliary tree.

Magnetic Resonance Cholangiopancreaticography (MRCP) was performed to further evaluate the biliary system and characterise the lesion. The mass, seen in the CT examination, was identified as a mixture of fat and soft tissue measuring 8.5cm x 6.3cm x 6.7cm. There was no communication between this mass and the biliary tree (Figures 3 and 4). The distal CBD was compressed by this mass.

Exploratory laparatomy was performed the same day and revealed a fatty encapsulated tumour lying below segment IV of the liver compressing the CBD. The hepatic artery was seen coursing through the mass, and had to be ligated before it was successfully resected *en mass* with the tumour and the atrophic left lobe of liver. The operating surgeons were of the opinion that the mass could represent an angioliposarcoma, because of its gross appearance.

The gallbladder was also removed. The rest of the liver, mesenteric vessels, spleen and bowels were normal. Gross pathologic examination showed the tumour mass attached externally to a piece of liver tissue, which was resected. The cut section showed fatty tissue, cystic areas containing sebaceous material and hair admixed with a firm fibrotic tissue. The resected hepatic artery was thick and fibrotic with a short course draining into the tumour.

The histology showed a mixture of mature adipocytes, hyalinised fibrous tissue, bony trabeculae and calcification. The attached liver showed cholestasis. The final pathologic diagnosis was mature benign teratoma. An ultrasound of the testes was performed and found to be normal. The patient was discharged and is currently on follow up at the surgical clinic.

## DISCUSSION

Teratomas are congenital neoplasms characterised by an abnormal growth of a combination of tissues derived from ectodermal, mesodermal and endodermal germ layers. This combination of tissues is unrelated to the organs where the tumour originates [1]. These primordial germ cells are initially detectable in the yolk sac of the four week embryo, and their migratory route during embryogenesis from the yolk sac to the gonads may account for the midline location of most extragonadal teratomas. They can be categorised as benign or malignant on the basis of their histopathological features.

These tumours mostly occur in the ovaries or the sacrococcygeal region in children. Less common anatomical locations include the testes, mediastinum and central nervous system. There have been rare cases of these tumours occurring in gastrointestinal tract, liver, nasal sinuses, cervix and thyroid [2].

Teratomas of the liver are rare neoplasms accounting for less than 1% of all teratomas. Even in paediatric patients where they are most commonly seen, they account for less than 1% of all liver neoplasms. In adults, benign teratomas do not directly affect the general condition of the patient; compression of the surrounding structures causes symptoms of abdominal distension, nausea and vomiting.

Occasionally acute abdominal pain can develop with malignant neoplasms, which are thought to cause rapid degeneration [3]. Hepatic teratomas are usually well encapsulated lesions and are easily resectable from the surrounding hepatic parenchyma [1], as was discovered by our surgeon.

In our patient, although the histology did not demonstrate tumour cells of hepatic origin, during the surgery, it was evident that the teratoma originated from the left lobe of liver in view of its common peritoneal attachments as well as capsular encasements. The CBD was compressed by the large tumour, which explained the occurrence of jaundice. The hepatic artery had to be ligated before it was resected *en masse* as it was encased within the tumour. A left hepatectomy was subsequently performed.

As previously mentioned, a teratoma is histologically composed of a variety of tissue elements. In our case, the histology revealed a mass containing solid adipose tissue, bony trabeculae, hair, fibrillary neural tissue and calcification.

Plain radiographs usually demonstrate a soft tissue mass with lucent fat opacity and calcifications, either rim like or chunky. An ultrasonography may demonstrate a hypoechoic (representing calcification or fat) or anechoic (representing the cystic portion of this mass) component.

Calcification demonstrates posterior acoustic shadowing. Bony tissue and calcification appear as high density foci on CT while fat is readily identified as hypodense with a HU of -60 to -100, demonstrating a density similar to subcutaneous fat. CT permits unequivocal identification of fat as adipose tissue and the characterisation of fluid as sebum, serous or complex. A component of a tumour that has both a CT attenuation value of fat and a horizontal interface with another more dependent type of fluid, most likely represents sebum [4].

In magnetic resonance imaging (MRI), calcification and bone produce no signal. Solid adipose tissue is of high signal intensity on both T1 and T2-weighted images. Fluid components are typically hypointense on T1 and hyperintense on T2-weighted images. The dermoid plug or Rokitansky protuberance is described by Anderson and Kissane [5] as an outgrowth from the inner surface of a cyst, containing hair and other atypical tissues and is one of the MRI findings of teratoma.

MRCP was useful in this case to demonstrate the compression of the CBD by the adjacent teratoma. Furthermore the delineation of the large vessels and the relationship of surrounding structures, as seen on the MRI was helpful in surgical planning, because all teratomas should be removed completely if possible, regardless of its benignity or malignancy.

In conclusion, the case described here represents a rare case of liver teratoma with interesting and characteristic CT and MRI correlation.
